# Minimally Invasive Arthroscopic Approach to Popliteal Tendon Impingement in a Degenerative Knee: A Case Report

**DOI:** 10.7759/cureus.83450

**Published:** 2025-05-04

**Authors:** Kei Nagasaki, Hiroki Ishikawa, Takuya Ohno, Taketoshi Seino, Manabu Mitsuhashi, Yoshifumi Kudo

**Affiliations:** 1 Department of Orthopedic Surgery, Showa University School of Medicine, Tokyo, JPN; 2 Department of Orthopedic Surgery, Nippon Koukan Hospital, Kawasaki, JPN

**Keywords:** arthroscopy, osteoarthritis, osteophyte, popliteus, tendon

## Abstract

Posterolateral knee pain can occur due to various causes, including meniscal tears, intra-articular loose bodies, ligament injuries, and tendinitis. Popliteal tendon impingement (PTI) due to osteophytes is a less common but significant cause of posterolateral knee pain, and treatment options for PTI are not well established. This report aimed to explore a minimally invasive approach to treating PTI in a degenerative knee using arthroscopic osteophyte resection. A 62-year-old man presented to our hospital with posterolateral knee pain and restricted range of motion (ROM) in the right knee. Imaging revealed significant osteophyte formation causing PTI. Conservative treatment failed. Thus, arthroscopic osteophyte resection was performed. Postoperatively, the patient significantly improved. The ROM improved from 90° of flexion with an extension lag of 5° preoperatively to 130° of flexion and full extension at three months postoperatively. The Knee Injury and Osteoarthritis Outcome Score (KOOS) improved from 52% to 74%, with complete resolution of pain. Comprehensive management, including precise diagnosis, judicious patient selection, and targeted arthroscopic intervention, is critical regardless of whether PTI is encountered in degenerative knees or following total knee arthroplasty (TKA). Arthroscopic osteophyte resection is a promising option for alleviating pain and improving function while preserving stability. Further studies are needed to strengthen the evidence base.

## Introduction

Posterolateral knee pain can be caused by various factors, including lateral meniscal tears, intra-articular free bodies, lateral collateral ligament injuries, and popliteus tendinitis [1]. Popliteal tendon impingement (PTI) due to osteophytes is a less common but significant cause of posterolateral knee pain. Optimal treatment of PTI has not been well established [1,2]. The popliteus tendon originates from the posterior aspect of the lateral femoral condyle, passes beneath the lateral collateral ligament, and inserts into the proximal posterior tibia [3]. In cases of popliteal impingement, osteophytes or anatomical abnormalities, such as a deep popliteal groove and prominent lateral femoral condyle components after total knee arthroplasty (TKA), can irritate the tendon, leading to snapping and mechanical stress [1,4-6]. Alleviating this mechanical irritation is crucial for managing impingement-related symptoms [5]. Despite being relatively rare, popliteal impingement can lead to significant functional impairment. Conservative management is often insufficient in cases of mechanical impingement, making surgical intervention necessary [7]. Osteophyte resection has been reported to yield positive outcomes in other joints, such as the shoulder and ankle [8,9]. Therefore, arthroscopic resection may be a minimally invasive treatment for PTI. This case report aimed to present a case of right knee osteoarthritis (OA) complicated by PTI due to osteophytes successfully treated with arthroscopic resection and illustrate the effectiveness of arthroscopic resection in resolving symptoms and improving knee function.

## Case presentation

**Figure 1:**
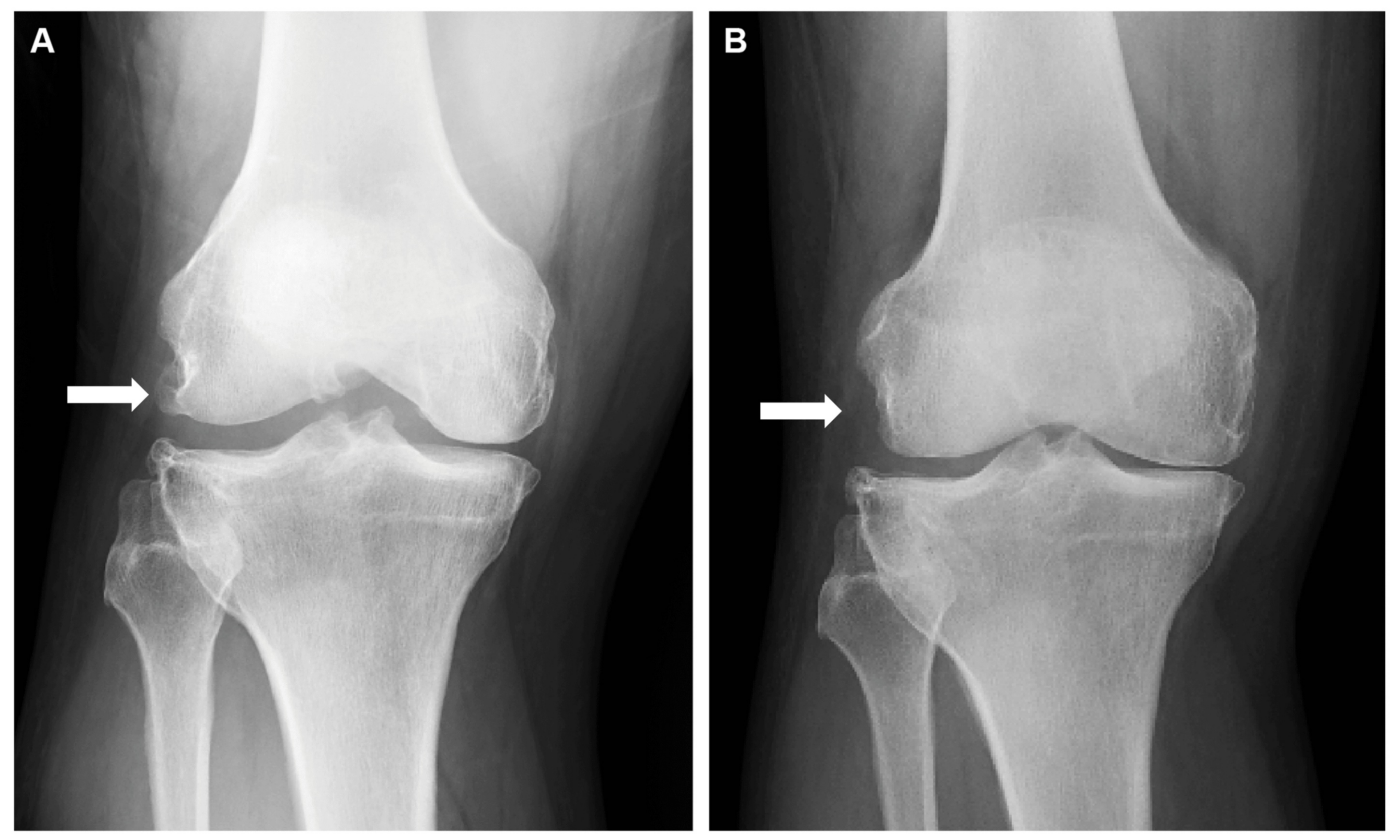
Preoperative (A) and postoperative (B) X-ray images of the right knee. The arrow in the preoperative X-ray shows a large osteophyte on the lateral femoral condyle that impinges on the popliteal tendon. The postoperative X-ray confirms the absence of an osteophyte following arthroscopic resection as indicated by the arrow. LFC: lateral femoral condyle

**Figure 2:**
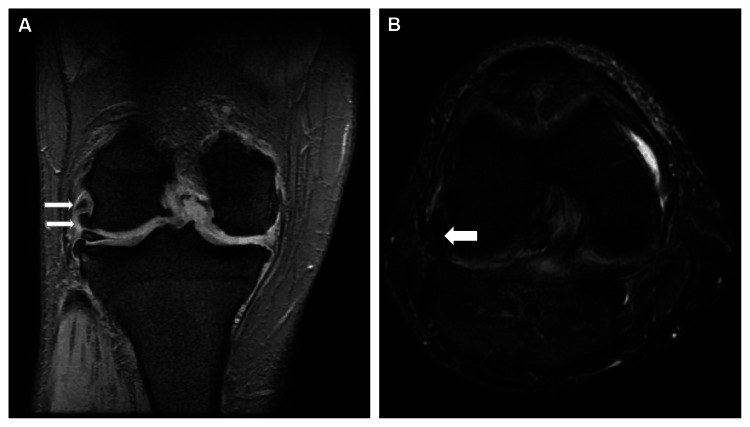
Postoperative MRI images of the right knee. (A) Coronal MRI slice. The upper white arrow indicates the popliteal tendon, which was impinged by the osteophyte, whereas the lower white arrow indicates the osteophyte on the lateral femoral condyle. (B) Axial MRI slice. The arrow indicates the osteophyte on the lateral femoral condyle, located immediately next to the popliteal tendon. LFC: lateral femoral condyle

A 62-year-old man with a history of right knee OA presented to our hospital with posterolateral pain and swelling in his right knee. Physical examination revealed a limited range of motion (ROM) in the right knee, with flexion restricted to 90° and an extension lag of 5°. Tenderness was noted on palpation along the lateral joint line. Radiographic imaging revealed diffuse OA with significant osteophyte formation on the lateral femoral condyle (Figure 1A). There was also a small osteophyte at the lateral edge of the tibia, while no osteophytes were observed in the posterior condyle or other joint compartments. Magnetic resonance imaging (MRI) confirmed the diagnosis of PTI caused by a large osteophyte, which was determined to be responsible for the patient’s symptoms (Figure 2).

**Figure 3:**
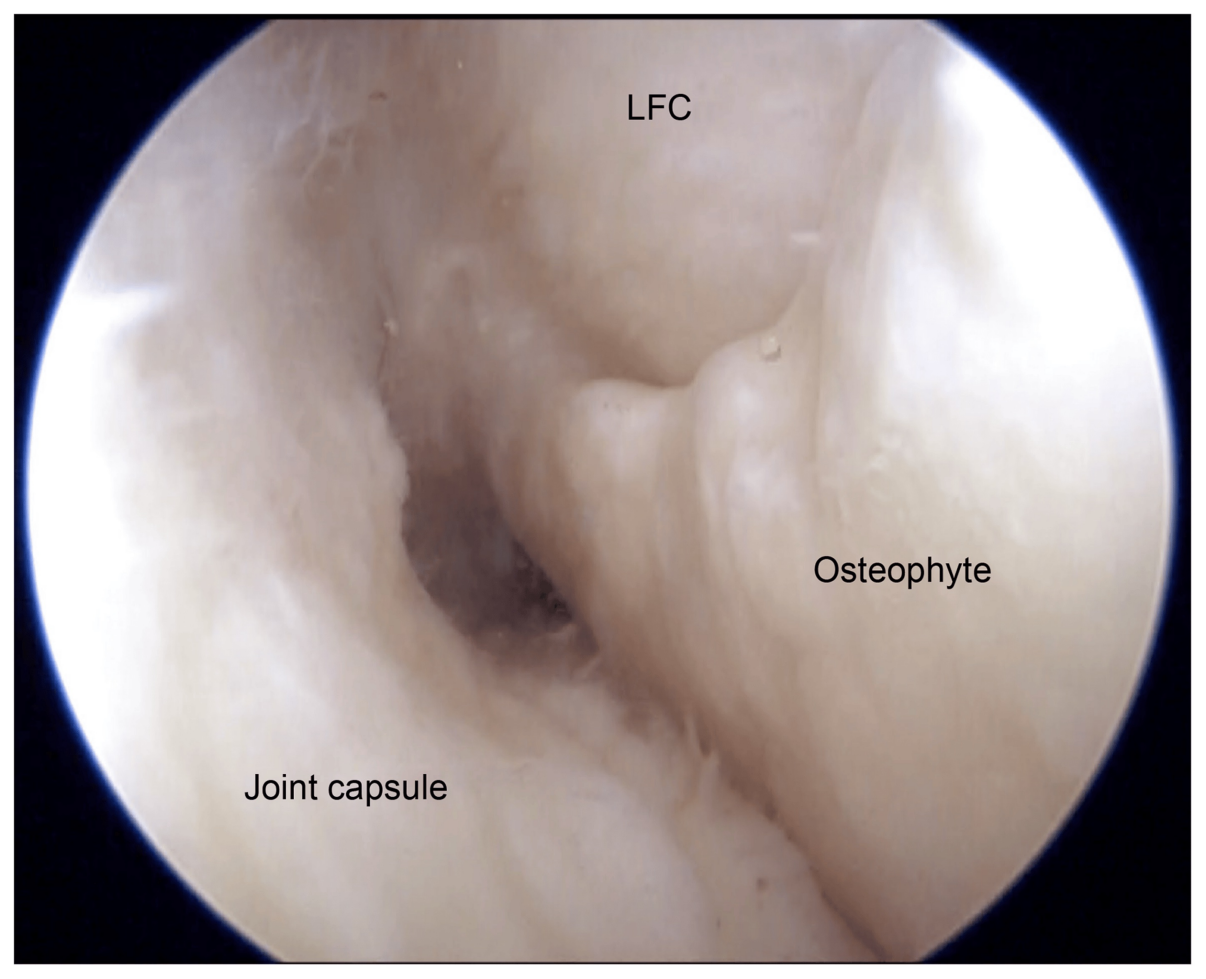
Arthroscopic view from the anterolateral portal of the right knee. Intraoperatively, a large osteophyte was observed in the lateral groove of the knee joint on the lateral femoral condyle. LFC: lateral femoral condyle

**Figure 4:**
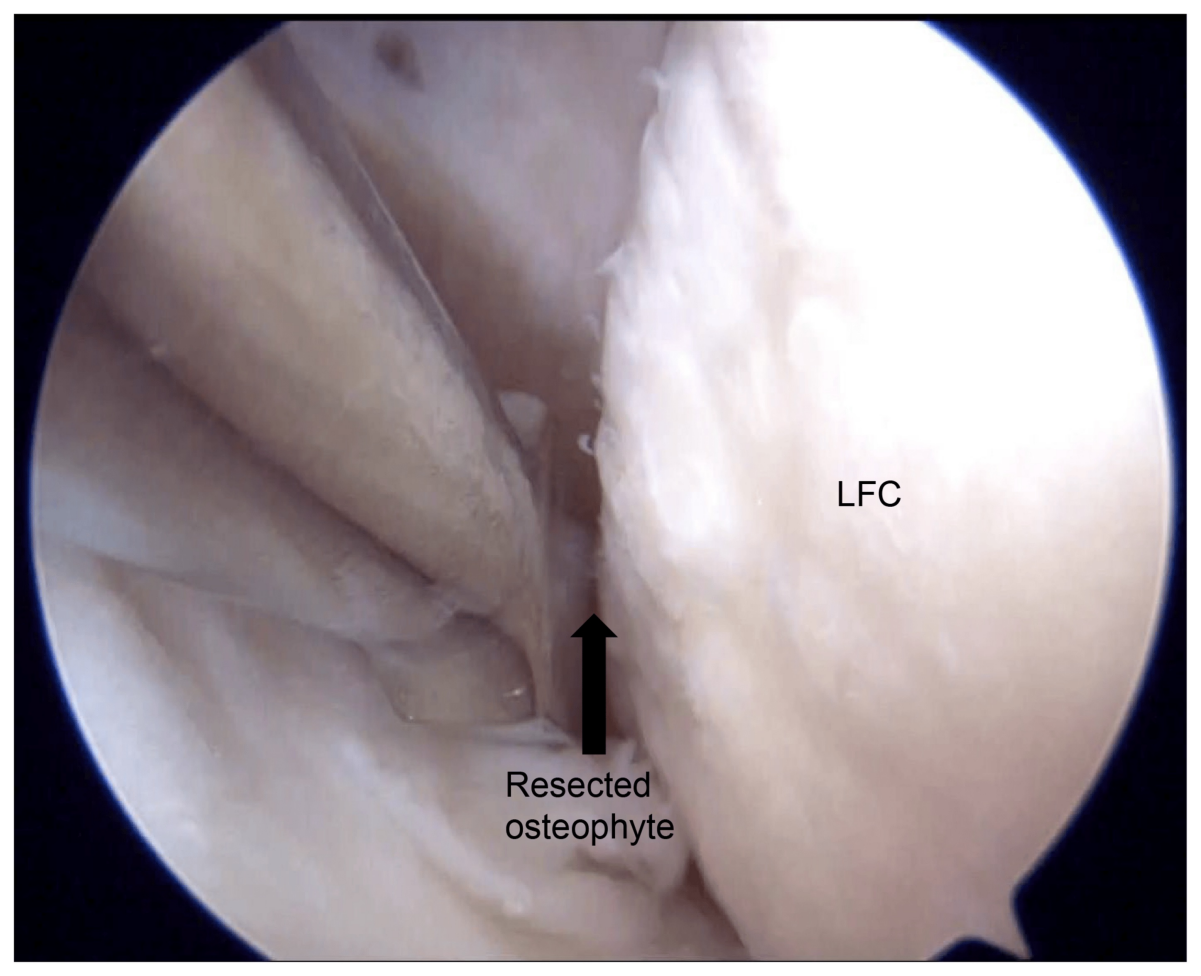
Arthroscopic view from the anterolateral portal of the right knee. Intraoperatively, an osteotome was used to resect the osteophyte from the lateral femoral condyle at the site where it impinged upon the popliteal tendon. LFC: lateral femoral condyle

**Figure 5:**
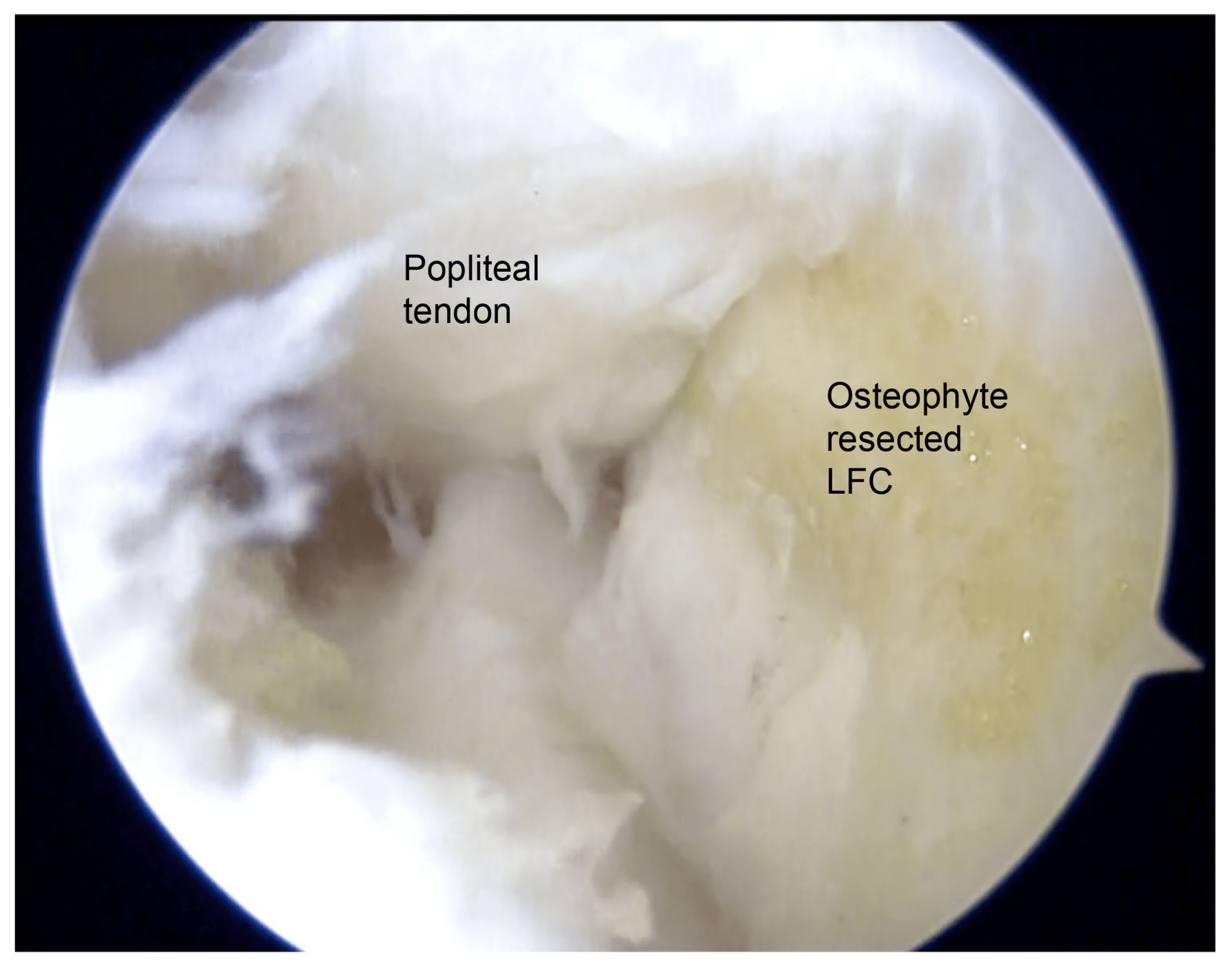
Arthroscopic view from the anterolateral portal of the right knee following osteophyte resection. The resected osteophyte measured approximately 7 mm in diameter. No further popliteal tendon impingement was observed at the lateral femoral condyle. LFC: lateral femoral condyle

Corticosteroid injection was administered to the affected area on an outpatient basis, but it yielded no symptomatic relief. Due to the progressive nature of the patient’s symptoms and confirmation of mechanical impingement, surgical intervention was planned. Arthroscopic osteophyte resection was performed using a third portal technique to access and remove the osteophyte near the popliteal tendon and under the lateral meniscus (Figure 3). Osteophyte resection of the lateral femoral condyle was performed using an osteotome and a mallet (Figure 4). Intraoperative findings confirmed the compression of the popliteal tendon by the osteophyte, which contributed to the patient’s limited ROM and pain (Figure 5).

The patient exhibited significant clinical improvement after surgery. At three months postoperatively, the ROM of the right knee improved to 130° of flexion with full extension, allowing for greater mobility. The Knee Injury and Osteoarthritis Outcome Score (KOOS), which was 52% preoperatively, significantly increased to 74% (Table 1), indicating a substantial reduction in pain and improvement in knee function. One of the most notable postoperative outcomes was the complete resolution of the posterolateral pain, which was attributed to PTI due to the distal femoral osteophyte. The patient was able to regain confidence in weight-bearing activities and daily movements.

**Table 1:** Changes in KOOS subscale scores. The KOOS includes five subscales. Each subscale is scored on a scale from 0 to 100, with higher scores indicating better knee function. “Total” represents the overall summary score. KOOS: Knee Injury and Osteoarthritis Outcome Score; ADL: activities of daily living; Sports/Rec: sports and recreation; QOL: quality of life

	Preoperation	Postoperation (3 months)	Postoperation (18 months)
Symptom	50	75	75
Pain	64	72	78
ADL	75	88	85
Sport/Rec	20	65	50
QOL	50	69	75
Total	52	74	73

At 18 months postoperatively, the KOOS remained stable at 73% (Table 1), indicating sustained functional benefits and pain relief. No recurrence of impingement-related symptoms was observed, and the patient could return to routine activities without significant discomfort or restriction. Overall, the surgical intervention (arthroscopic osteophyte resection) successfully alleviated the primary symptoms and contributed to long-term functional recovery, thereby improving the patient’s quality of life.

## Discussion

In this case, the successful treatment of osteophyte-induced PTI indicates that arthroscopic osteophyte resection is an effective approach to managing mechanical complications arising from advanced OA. Although osteophyte formation and cartilage degeneration associated with OA progression are widely recognized, secondary complications such as PTI caused by osteophytes have not received sufficient attention [1,2]. PTI is generally considered a complication after TKA [6,10]. However, it can also occur in degenerative knees, as demonstrated in our case. A recent systematic review of eight studies involving 26 cases reported multiple contributory factors, including component size, component positioning, and osteophyte-induced mechanical conflict [7]. These findings indicate that osteophyte-related impingement can occur in non-TKA scenarios, highlighting the importance of meticulous preoperative imaging and prudent assessment.

Imaging modalities, such as ultrasound, computed tomography, and MRI, identified sizable osteophytes that caused mechanical obstruction. Furthermore, ultrasound-guided diagnostic injections confirmed PTI with high reproducibility, indicating a broader applicability beyond TKA [11]. In our case, conservative treatment failed to alleviate the posterolateral pain and functional impairment. Surgical intervention is an option when conservative approaches are unsuccessful [10]. In this case, arthroscopic resection of the offending osteophytes was performed using a third portal, which resulted in significant improvements in pain, ROM, and overall function. The KOOS increased from 52% to 74% at three months postoperatively (Table 1), and the posterolateral pain completely resolved. No postoperative instability or adverse events occurred, which is consistent with the findings of an existing study that supports the safety and efficacy of arthroscopic techniques [12].

Meanwhile, the potential impact of popliteus tendon release or tenotomy on knee stability, especially in TKA, remains a concern [4]. Although some studies have described laxity after popliteus tendon release in cruciate-retaining or posterior-stabilized prostheses, no study has reported any instability or adverse outcomes [4,7]. Additionally, the diagnosis of PTI depends on recognizing localized posterolateral knee pain rather than diffuse discomfort [10]. Diagnostic injections can be of great utility when imaging alone is inconclusive, and intraoperative findings, such as a “snapping” tendon during ROM testing, may guide the diagnosis [10,11]. Partial tendon release, if necessary, does not appear to compromise clinical outcomes [4,5]. Arthroscopic osteophyte resection is a minimally invasive procedure that can reduce postoperative discomfort and facilitate quicker recovery [10]. This case demonstrated that early removal of localized mechanical impingement might delay the need for more extensive surgeries, such as TKA (Figure 1B). However, long-term follow-up is essential for monitoring osteophyte recurrence. In case of osteophyte formation recurrence, additional surgical procedures may be required, or the patient may eventually progress to TKA if conservative management becomes ineffective.

## Conclusions

A comprehensive approach that includes accurate diagnosis, careful patient selection, and targeted surgical intervention is required for the management of PTI, whether in the context of OA or post-TKA. Arthroscopic osteophyte resection is a safe and effective treatment option, particularly for patients with localized impingement and mechanical symptoms. Although further studies and long-term follow-up are needed to establish high-level evidence and efficacy of this procedure as a standard treatment option, our case demonstrates that addressing the mechanical causes of PTI can lead to significant pain relief and functional recovery.
